# Seven quick tips for analysis scripts in neuroimaging

**DOI:** 10.1371/journal.pcbi.1007358

**Published:** 2020-03-26

**Authors:** Marijn van Vliet

**Affiliations:** Department of Neuroscience and Biomedical Engineering, Aalto University, Espoo, Finland; Dassault Systemes BIOVIA, UNITED STATES

## Introduction

In neuroimaging, as in many fields, the journey of the data from the measurement equipment to a figure in a publication is growing longer and more complicated. New preprocessing steps are added at the beginning of the pipeline [1–3], new multivariate techniques in the middle [4–6], and new statistical methods at the end [7]. Using these new techniques often requires writing pieces of programming code referred to as "scripts," and, in accordance with the growing data analysis pipelines, these scripts also tend to increase in length and complexity. When programming code becomes sufficiently convoluted, even the most experienced programmers will make mistakes, which can ultimately lead to erroneous conclusions. Papers have had to be retracted due to programmer error [8–10].

Ideally, analysis code should be a testament to the scientific rigor and technological creativity of the researcher, in much the same way as the scientific articles they write. This means dealing with the disorder that arises as ideas get tried and discarded, mistakes are made and fixed, and smaller analyses are made on the side. The tips in this article aim to facilitate a successful organization of the analysis code, thereby keeping the complexity of data analysis code within tolerable limits and reducing errors.

This article is a guide for those who have already written their own data analysis pipelines and wish to improve their designs. Those looking for information on how to get started writing a data analysis pipeline for neuroimaging are referred to other works [11–15].

There is a large body of literature on managing complexity and reducing the chance for programmer error in software [16–20]. However, most of it deals with the construction of programs and software libraries intended to be used across projects. This article focuses on analysis scripts that sit at the point at which the general purpose functionality that is exposed by software libraries meets the specific demands of a single study [21]. This makes scripts somewhat of an outlier in the software landscape, as they are pieces of code that are not reusable (pieces that need to be reusable are better implemented in a software library), only have to function correctly on one specific data set (hence there is little need to test for "edge cases"), and will generally only be used by yourself and your collaborators (save the occasional run for review purposes and replication studies). Therefore, many of the standard practices of the software industry do not apply or need translation in order to arrive at concrete advice of what to do and what to avoid when writing analysis scripts.

To move beyond mere truisms, the analysis pipeline developed by van Vliet and colleagues [22] has been extended to implement all tips in this paper and will be used as case study to discuss the practical consequences of each tip in terms of code. The example pipeline starts from the raw magnetoencephalography (MEG) and structural magnetic resonance imaging (MRI) data from the Wakeman and colleagues [23] data set and performs several artifact reduction steps, source estimations, functional connectivity analyses, cluster permutation statistics, and various visualizations. The size and complexity of the pipeline is representative of that of the pipelines in modern studies at the time of writing. Where van Vliet and colleagues [22] give a detailed explanation of all analysis steps, the current paper focuses on the design decisions that were made during the implementation. You can find the code repository for the analysis pipeline at: https://github.com/aaltoimaginglanguage/conpy. Of special interest is the scripts folder of that repository, which contains the analysis scripts. The "Application of the tip to the example analysis" sections refer frequently to the example code, and it is recommended to study these sections and the code side by side. The electronic version of this document contains many hyperlinks to sections of the code, which the reader is encouraged to follow to see how the tips can be implemented in practice.

By nature, analysis scripts are specific to a single study. Hence, the primary intention for the example pipeline is to be a source of ideas to use when writing your own analysis pipelines. However, should you wish to construct a pipeline similar to the example, a stripped down version can be found at https://github.com/aaltoimaginglanguage/study_template along with instructions on how to use it as a template for new analysis pipelines.

## Tip 1. Each analysis step is one script

An effective strategy to reduce software complexity is to break up a large system into smaller parts [24, 25]. The first tip is therefore to isolate each single step of an analysis pipeline into its own self-contained script. This greatly reduces complexity by allowing us to reason about the pipeline on two levels. At the lower level, we can reason about the implementation of a single step, while ignoring the rest of the pipeline for a moment. At the higher level, we can treat the individual steps as "black boxes" and focus on how they are combined together to form the complete pipeline (see also Tip 3), ignoring their implementation for a moment.

This raises the question of what exactly constitutes a single analysis step. The decision of where to "cut" the pipeline can be made from different perspectives:

### Complexity perspective

Each individual script should be easy to understand and reason about, so one way to define a single step is by its complexity: If a single script becomes too complex to be easily understood as a whole, it should be split up into smaller steps, if possible.

### Thematic perspective

Ideally, understanding one script should not require knowledge of another script. If each script can be viewed as a self-contained box that performs a single task, the pipeline as a whole becomes simply a collection of these boxes that are executed in a specific order. A script should therefore aim to implement a single task (not multiple) and implement it completely, not only part of it. See Tip 2 for another thematic consideration.

### Time perspective

While running the entire analysis pipeline may take days, a single script should finish in a reasonable time. This invites frequent testing as you iteratively develop the script, allows you to quickly evaluate the effect of a parameter, and makes it painless to ensure that the latest version of the script matches the latest result.

These perspectives may clash with another, so compromises are sometimes necessary. We will now look how these perspectives influenced the way the van Vliet and colleagues’ [22] pipeline was split into individual steps.

### Application of the tip to the example analysis

**Fig 1:**
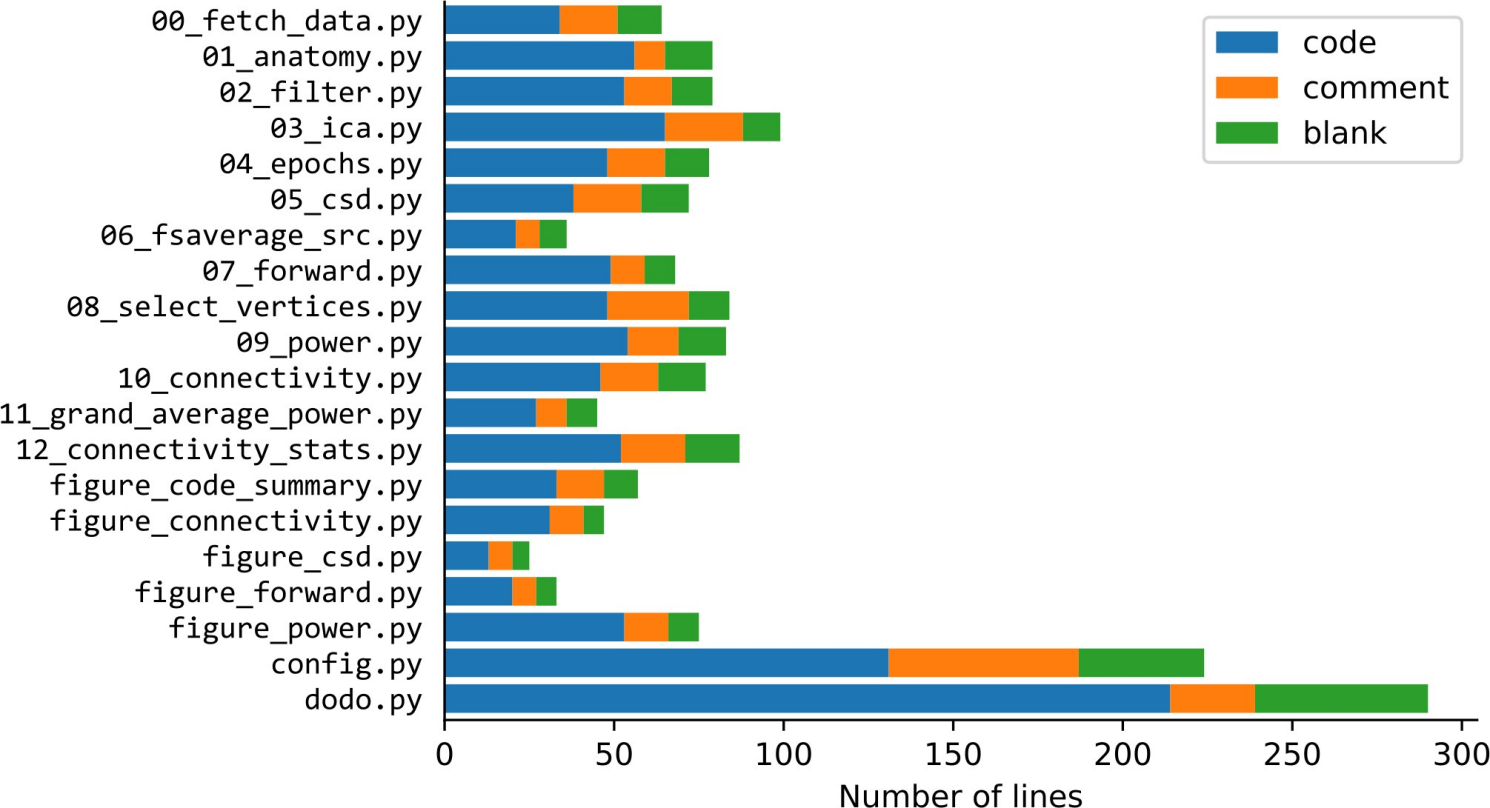
For each script in the analysis pipeline, the number of lines of the file, broken down into lines of programming code (code), lines of descriptive comments (comment), and blank lines (blank). The first 13 scripts perform data analysis steps, the next five scripts generate figures, the config.py script contains all configuration parameters, and the dodo.py script is the master script that runs all analysis steps on all recordings.

The van Vliet and colleagues [22] pipeline consists of 13 analysis scripts that process the data, five visualization scripts that construct the figures used in publications, a configuration file (config.py), and a "master" script that calls the individual analysis scripts (dodo.py, see Tip 3). The analysis scripts are relatively short (Fig 1), containing an average of 40.8 lines of code (SD 14.8), while the configuration (see Tip 6) and master scripts (see Tip 3) are longer.

Often, the reasoning behind the scope of each script was made from a thematic perspective. For example, one script performs the source estimation (09_power.py) and another the connectivity estimation (10_connectivity.py). However, the decision to split the artifact reduction steps into two scripts (02_filter.py; 03_ica.py) was made from a time perspective. Since the independent component analysis (ICA) computation takes time, it was split off into its own script to avoid having to repeat it unnecessarily. Finally, the decision to split up the construction of the forward models (i.e., leadfield) into three steps (06_fsaverage_src.py, 07_forward.py, 08_select_vertices.py) was made from a complexity perspective.

## Tip 2. A script either processes a single recording or aggregates across recordings, never both

In neuroscience, it is common to apply the same data processing steps to multiple recordings. For example, a frequently seen construct is the “big loop” over data from multiple participants. However, running a single processing step on a single recording is a very common use case (for example, during development or when a problem is encountered with one of the recordings), which the design should make possible without having to modify (e.g., "commenting out") any code.

Hence, the second tip is that scripts should generally only operate on a single recording, passed as a parameter from the command line, and not have the “big loop” (instead, the script is applied to all recordings in a separate "master" script [see Tip 3]). The exceptions to this are scripts that aggregate data across recordings (e.g., compute a grand average or perform statistics) and therefore must have the “big loop,” in which case the sole purpose of the script should be aligning the data (e.g., morphing to a template brain) and computing the aggregate. This reduces the complexity of the code, since it allows the reader to either focus on the intricacies of a data processing step (without having to worry about how the data is later reconciled across recordings) or focus solely on the details of how multiple data sets are aligned and combined.

### Application of the tip to the example analysis

In the example pipeline, there is a strict separation between scripts that perform data analysis on a single participant (steps 0–10) and scripts that aggregate across participants (steps 11 and 12). The scripts implementing steps 0–10 all take a single command line parameter indicating the participant to process. This is implemented with the argparse module of the standard Python library, which facilitates the generation of a helpful error message when this parameter is omitted, along with documentation on how to run the script. Not only does this help to keep the number of lines of code and the running time of the script down (Fig 1), it also opens up the possibility for the master script (see Tip 3) to automatically skip running the script for participants that have already been processed earlier.

## Tip 3. One master script to run the entire analysis

Once the individual steps have been implemented as a collection of scripts, the pipeline can be assembled in a "master" script that runs all the steps on all the data. This master script is the entry point for running the entire analysis, and therefore the third tip is that there should ideally only be one such script.

By having a strict separation between the scripts that implement the individual steps and the master script, it becomes possible to view the pipeline on two levels: The implementation details of each single step and how the steps fit together to build the pipeline. To understand the latter, the master script provides the "floor plan" of the analysis, which can be studied without having to go into detail on how each individual step is performed. Hence, the only function that the master script should perform is to call the other scripts in the correct order. Actual data analysis steps, including logic for combining results across scripts, should always be performed in a separate script, which is in turn called from the master script.

A master script can be as simple as having a single script that executes all other analysis scripts in order. However, if some analysis steps take a long time to run, it becomes worthwhile to implement functionality to make sure an analysis script is only run when needed. Keeping track of which scripts have changed since they were last ran and whether the input data to a script has changed is a task that has been studied in great detail in the area of software engineering, and many specialized tools, known as "build systems," are available to perform the required bookkeeping tasks. For example, build systems that are optimized for creating data analysis pipelines include snakemake, pydoit, luigi, and nipype [26].

### Application of the tip to the example analysis

**Fig 2:**
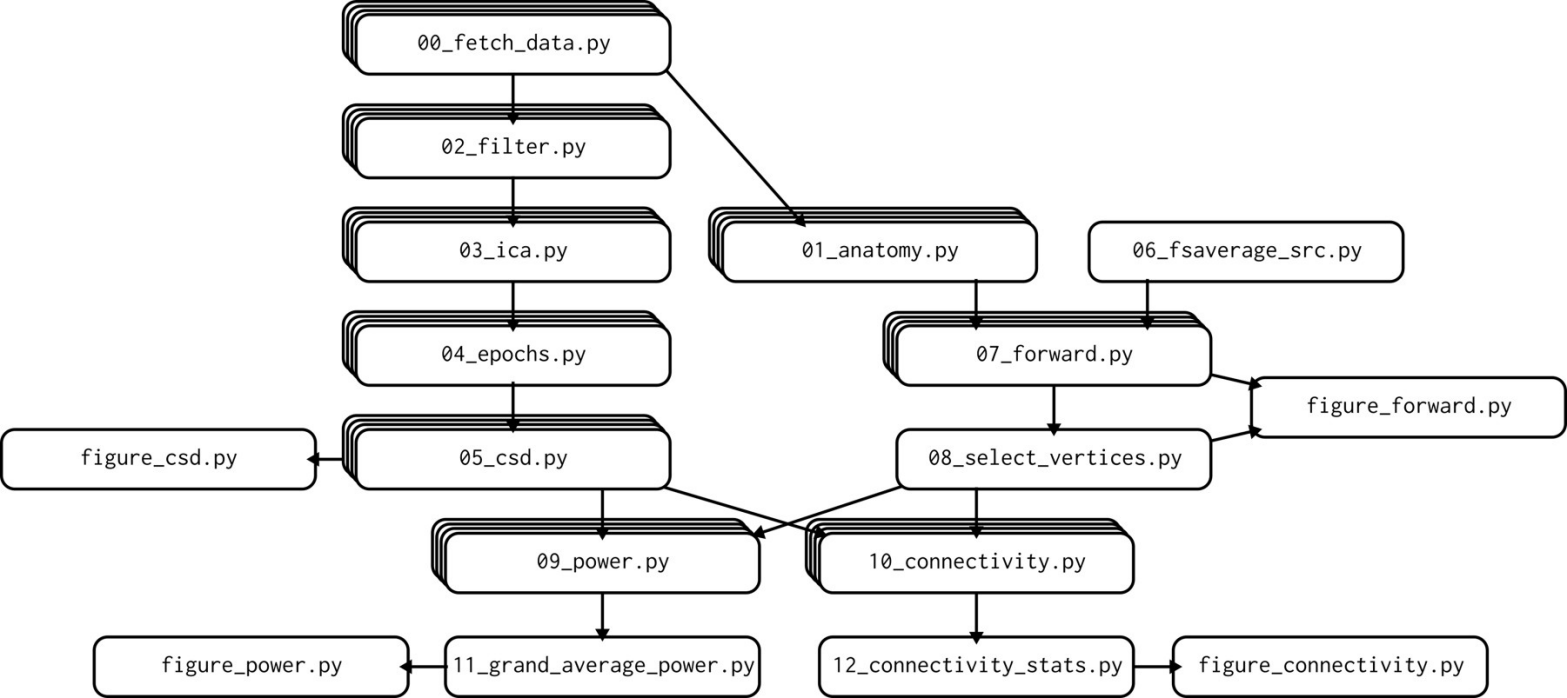
Dependency graph showing how the output of one script is used by another. Stacked boxes indicate scripts that are run for each participant.

The master script of the example pipeline, dodo.py, is implemented using the pydoit build system. In the script, all analysis steps are described as "tasks", with steps 0–10 having a "subtask" for each participant. Each task is associated with one of the analysis scripts, along with a list of files that are used by the script and produced. This allows the build system to work out the dependency graph of the analysis pipeline (Fig 2).

The build system keeps track of which tasks are "up to date," which means the latest version of the analysis scripts of all analysis steps up to and including the current step have all been run. This means that the entire analysis pipeline can be run often and cheaply: All steps that are up to date will be skipped. Making a change anywhere within the analysis code will prompt the recomputation of all the steps that need to be rerun.

The build system also provides a set of commands that allow for executing specific parts of the pipeline (e.g., a single analysis step on all participants, a few specific steps on a few specific participants, etc.) without having to change (e.g., comment out) any code.

## Tip 4. Save all intermediate results

The fourth tip is that all intermediate results generated during the execution of a script should be stored, if feasible.

From a complexity perspective, the more each script can be isolated from the rest of the pipeline, the easier it is to understand and represent as a self-contained black box. Hence, it is important that each script can function in isolation and does not rely on data that was left in memory by another script. Another big advantage is that saving intermediate results makes it possible to skip any data processing steps that are unchanged since the last time the pipeline was executed (see also Tip 3). Finally, having all intermediate results readily available facilitates manual checks and exploration of the data.

In cases of limited storage capability, the cost of storage needs to be weighed against the benefits of not having to recompute the result. Here, it helps to identify computational bottlenecks and only store the minimum amount of data needed to bypass the bottleneck when rerunning the script. For example, for linear data transformations (such as principal component analysis [PCA], ICA, minimum norm esitimates [MNE], and beamformers) the computational bottleneck is in the computation of the transformation matrix, which by itself does not take much disk space. Applying the transformation matrix to the data is computationally cheap, while the result may take up a large amount of disk space.

### Application of the tip to the example analysis

In the example analysis pipeline, each script begins by loading the data that were produced by previous analysis steps as requires. Each script ends by saving all data that was produced by the script. This includes the processed MEG data and also, for example, the ICA decomposition matrix, along with the indices of the ICA components that were judged to correspond to eye-blink contaminants.

## Tip 5. Visualize all intermediate results

Data analysis pipelines, such as those used in neuroscience, are sufficiently complex that failures should be expected and planned for. When designing the pipeline, think about the system for catching errors when they happen.

While using analysis scripts offers many advantages over using a graphical user interface (GUI), a severe disadvantage is the lack of direct feedback. Since the result is usually not immediately visualized, errors may stay hidden for a long time. Programming a computer is not unlike receiving a wish from a mischievous genie: You will get exactly what you asked for but not necessarily what you wanted. As long as the final result of a series of processing steps looks reasonable, intermediate steps might contain nonsensical results that we would never know about unless we take care to check everything. Therefore, an analysis pipeline should invite frequent visual checks on all intermediate results.

The fifth tip is that for each intermediate result, the script should create a visualization of the result and save it to disk. This does not need to be a publication ready figure, but must provide a visual confirmation that the data analysis operation had the intended result. By re-creating the figures every time a script is run and overwriting the file on disk, the figure remains up to date.

After running all the analysis scripts, a complete visual record should be available of the data as it moves through the pipeline. Care should be taken that the order of the figures matches that of the analysis steps. Such a record invites frequent visual checks of the obtained results and therefore somewhat offsets the main advantage that GUI programs have over scripting.

### Application of the tip to the example analysis

Whenever an intermediate result is saved to disk in the example analysis pipeline, a simple visualization is also created and added to a "report" file. The main analysis package used in the example pipeline, MNE-Python [27], provides a report class that compiles a set of figures into a single HTML file. Each script adds (and overwrites) figures to the same report, which will grow in length as more scripts are run. The resulting HTML file contains an easy to navigate visual record of the data flowing through the pipeline. Each participant has their own report file.

## Tip 6. Each parameter and file name is defined only once

It is not uncommon that a parameter is used in multiple scripts. The sixth tip is that the value of each parameter should be defined in one place. Instead of copying the value of a parameter into all scripts that need it, the parameter should be imported, i.e., the programmer specifies the location where the parameter is defined and the programming language will take care of fetching the value when it is needed. In the programming literature, this is referred to as the "don't repeat yourself" (DRY) principle [18].

A good tactic for managing parameters is to create a single configuration script, which sole function is to define the values for all parameters. It makes it obvious where to look for the definition of a parameter and decreases the chances of accidentally defining the same parameter at two different locations.

File names are also parameters and ones that are commonly shared across scripts too. One script produces a file that is used by another. Just like other parameters, the tip mandates that all file names should be defined once and imported (not copied) by scripts that need it. It is not uncommon for a file name to change when parts of the analysis pipeline are added or removed. By ensuring the change needs to be made in only one location, scripts are less likely to load data from the wrong file.

### Application of the tip to the example analysis

The example pipeline has a central configuration script config.py, which defines all relevant parameters for the analysis, such as filter settings, the list of subjects, the experimental conditions, and so forth. All analysis scripts import the configuration file and thereby gain access to the parameters. Whenever a parameter needs to be changed or added, the configuration file is the single authoritative location where the edit needs to be made, and the change is propagated to all analysis scripts.

The config.py script starts by offering some machine specific parameters, such as the number of central processing unit (CPU)-cores to dedicate to the analysis and where on the disk the data is to be stored. The configuration script queries the hostname so that different parameters can be specified for different machines.

Since the pipeline stores all intermediate results and their visualizations, there is a large number of file names to deal with. In many cases, each file name is used four times: Once in the script generating the file, once in the script consuming the file, and twice in the master script, dodo.py. For this reason, a helper class, Filenames, has been written that offers an efficient way to manage them. The class is used to create an fname namespace that contains short aliases for all file names used throughout the pipeline. It also leverages Python's native string formatting language to allow quick generation of lists of file names that adhere to a pattern (e.g., "sub01_raw" and "sub02_raw").

## Tip 7. Distinguish files that are part of the official pipeline from other scripts

The development of a complex analysis pipeline is seldom a straightforward path from start to finish, and the creative process thrives under a certain level of messiness [28]. However, when miscellaneous files are scattered about (littering the folders that contain the main pipeline), they obfuscate what is relevant and what is not. A good strategy is to alternate between bouts of creativity (where "technical debt" [29] is allowed to accumulate) and periods of cleaning up, during which the overall organization and design of the pipeline is reevaluated and the technical debt repaid.

The seventh tip calls for an organization system that distinguishes between files that are in a stable state and part of the main pipeline and files that are work in progress, temporary, or part of analyses on the side. A good system reduces the effort of the cleaning process, making it easier to commit to a regular tidying up of the virtual workplace. It is important that the system does not become burdensome, as a simple system that is actually used is better than a more powerful one that is not.

This can be implemented in whatever way suits your workflow best, ranging from simply maintaining a rigid naming convention and folder structure to using more powerful tools such as a version control system (VCS) [30, 31]. Note that a VCS is not an organization system in itself but merely a tool for implementing one. It is up to the data analyst to devise their own system and have the discipline to stick to it.

### Application of the tip to the example analysis

During the development of the pipeline, many scripts were written to try out different analysis approaches, conduct tests, and do miscellaneous other tasks. The naming system was such that all analysis scripts that are officially part of the pipeline are either prefixed with a number (00_– 12_) and all scripts that produce figures for the manuscript with figure_. From time to time, any script lacking such a prefix would be closely scrutinized to determine whether is was still relevant and (if not) deleted. Deleted files were never truly gone though, as the project is managed by the VCS git [31, 32]. Git keeps track of the history of a file, allowing to return to previous versions, as well as parallel copies when doing something experimental.

## Conclusion

Writing understandable code is a skill that can be honed by forming good habits. Whenever a problem arises in a pipeline, there is an opportunity to look beyond the specific problem to the circumstances that allowed the problem to occur in the first place and the formation of new habits to prevent such circumstances in the future. However, it is important not to become bogged down in rules. Every new project is a chance for reviewing your habits: Keep things that were beneficial and drop things that were not or which costs exceed their utility.

Be aware that there is a lot of software tooling available to automate repetitive tasks and perform bookkeeping. Whenever a rule needs enforcing or a repetitive action needs performing, there is likely a software tool available to automate it. However, while they can make it easier to adopt good habits and keep the code organized, they cannot do the job by themselves. Ultimately, it is up to the data analyst to keep things tidy and reevaluate the design of the analysis pipeline as it grows. When and how to do this is best learned through experience. By reflecting at the end of each project what were good and bad design choices, the analysis pipeline of the next project will be better than the last.

## References

[1] Bigdely-Shamlo N, Mullen T, Kothe C, Su KM, Robbins KA. The PREP Pipeline: Standardized Preprocessing for Large-Scale EEG Analysis. Frontiers in Neuroinformatics. 2015;9(June):16 10.3389/fninf.2015.00016 26150785 PMC4471356

[2] Nolan H, Whelan R, Reilly RB. FASTER: Fully Automated Statistical Thresholding for EEG Artifact Rejection. Journal of Neuroscience Methods. 2010;192(1):152–162. 10.1016/j.jneumeth.2010.07.015 20654646

[3] Jas M, Engemann DA, Bekhti Y, Raimondo F, Gramfort A. Autoreject: Automated Artifact Rejection for MEG and EEG Data. NeuroImage. 2017;159:417–429. 10.1016/j.neuroimage.2017.06.030 28645840 PMC7243972

[4] Kriegeskorte N, Mur M, Bandettini P. Representational Similarity Analysis—Connecting the Branches of Systems Neuroscience. Frontiers in systems neuroscience. 2008;2(November):4 10.3389/neuro.06.004.2008 19104670 PMC2605405

[5] King JR, Dehaene S. Characterizing the Dynamics of Mental Representations: The Temporal Generalization Method. Trends in Cognitive Sciences. 2014;18(4):203–210. 10.1016/j.tics.2014.01.002 24593982 PMC5635958

[6] McIntosh AR, Mišić B. Multivariate Statistical Analyses for Neuroimaging Data. Annual Review of Psychology. 2013;64(1):499–525. 10.1146/annurev-psych-113011-143804 22804773

[7] Maris E, Oostenveld R. Nonparametric Statistical Testing of EEG- and MEG-Data. Journal of Neuroscience Methods. 2007;164(1):177–190. 10.1016/j.jneumeth.2007.03.024 17517438

[8] Casadevall A, Steen RG, Fang FC. Sources of Error in the Retracted Scientific Literature. The FASEB Journal. 2014;28(9):3847–3855. 10.1096/fj.14-256735 24928194 PMC5395722

[9] Miller G. Scientific Publishing. A Scientist's Nightmare: Software Problem Leads to Five Retractions. Science (New York, NY). 2006;314(5807):1856–1857. 10.1126/science.314.5807.1856 17185570

[10] Merali Z. Computational Science: …Error …why Scientific Programming Does Not Compute. Nature. 2010;467(7317):775–777. 10.1038/467775a 20944712

[11] Jas M, Larson E, Engemann DA, Leppäkangas J, Taulu S, Hämäläinen M, et al A reproducible MEG/EEG group study with the MNE software: recommendations, quality assessments, and good practices. Frontiers in Neuroscience. 2018;12 10.3389/fnins.2018.00530 30127712 PMC6088222

[12] Popov T, Oostenveld R, Schoffelen JM. FieldTrip made easy: an analysis protocol for group analysis of the auditory steady state brain response in time, frequency, and space. Frontiers in Neuroscience. 2018;12 10.3389/fnins.2018.00711 30356712 PMC6189392

[13] Tadel F, Bock E, Niso G, Mosher JC, Cousineau M, Pantazis D, et al MEG/EEG group analysis with Brainstorm. Frontiers in Neuroscience. 2019;13 10.3389/fnins.2019.00076 30804744 PMC6378958

[14] Andersen LM. Group analysis in MNE-Python of evoked responses from a tactile stimulation paradigm: a pipeline for reproducibility at every step of processing, going from individual sensor space representations to an across-group source space representation. Frontiers in Neuroscience. 2018;12 10.3389/fnins.2018.00006 29403349 PMC5786561

[15] Andersen LM. Group analysis in FieldTrip of time-frequency responses: a pipeline for reproducibility at every step of processing, going from individual sensor space representations to an across-group source space representation. Frontiers in Neuroscience. 2018;12 10.3389/fnins.2018.00261 29765297 PMC5938406

[16] Beck K. Test Driven Development: By Example. Addison-Wesley; 2002.

[17] Hunt A, Thomas D. The Pragmatic Programmer: From Journeyman to Master. Boston: Addison-Wesley Professional; 1999.

[18] Martin RC. Clean Code: A Handbook of Agile Software Craftmanship. Prentice Hall; 2008.

[19] McConnell S. Code Complete: A Practical Handbook of Software Construction. 2nd ed. Microsoft Press; 2004.

[20] Wilson G, Aruliah DA, Brown CT, Hong NPC, Davis M, Guy RT, et al Best Practices for Scientific Computing. PLoS Biol. 2014;12(1):e1001745 10.1371/journal.pbio.1001745 24415924 PMC3886731

[21] Schwen LO, Rueschenbaum S. Ten Quick Tips for Getting the Most Scientific Value out of Numerical Data. PLoS Comput Biol. 2018;14(10):e1006141 10.1371/journal.pcbi.1006141 30307934 PMC6181270

[22] van Vliet M, Liljeström M, Aro S, Salmelin R, Kujala J. Analysis of Functional Connectivity and Oscillatory Power Using DICS: From Raw MEG Data to Group-Level Statistics in Python. Frontiers in Neuroscience. 2018;12 10.3389/fnins.2018.00586 30271317 PMC6146299

[23] Wakeman DG, Henson RN. A Multi-Subject, Multi-Modal Human Neuroimaging Dataset. Scientific Data. 2015;2:150001 10.1038/sdata.2015.1 25977808 PMC4412149

[24] Hofmann MA. Criteria for Decomposing Systems Into Components in Modeling and Simulation: Lessons Learned with Military Simulations. Simulation. 2004;80(7–8):357–365. 10.1177/0037549704049876

[25] Parnas DL. On the Criteria to Be Used in Decomposing Systems into Modules. Communications of the ACM. 1972;15(12):1053–1058. 10.1145/361598.361623

[26] Gorgolewski KJ, Esteban O, Ellis DG, Notter MP, Ziegler E, Johnson H, et al Nipy/Nipype: Release 0.13.1. 2017; 10.5281/zenodo.581704

[27] Gramfort A, Luessi M, Larson E, Engemann DA, Strohmeier D, Brodbeck C, et al MEG and EEG Data Analysis with MNE-Python. Frontiers in Neuroscience. 2013;7(December):1–13. 10.3389/fnins.2013.00267 24431986 PMC3872725

[28] Mehta R, Dahl DW. Creativity: Past, Present, and Future. Consumer Psychology Review. 2019;2(1):30–49. 10.1002/arcp.1044

[29] Kruchten P, Nord RL, Ozkaya I. Technical Debt: From Metaphor to Theory and Practice. IEEE Software. 2012;29(6):18–21. 10.1109/MS.2012.167

[30] Blischak JD, Davenport ER, Wilson G. A Quick Introduction to Version Control with Git and GitHub. PLoS Comput Biol. 2016;12(1):e1004668 10.1371/journal.pcbi.1004668 26785377 PMC4718703

[31] Vuorre M, Curley JP. Curating Research Assets: A Tutorial on the Git Version Control System. Advances in Methods and Practices in Psychological Science. 2018;1(2):219–236. 10.1177/2515245918754826

[32] Chacon S, Straub B. Pro Git. 2nd ed. The Experts Voice; 2019.

